# Effectiveness of Korean medicine treatments in improving cognitive function and prefrontal cortex activity in older individuals with mild cognitive impairment: retrospective observational study

**DOI:** 10.3389/fneur.2024.1440111

**Published:** 2024-10-30

**Authors:** Yujin Choi, Kyungseok Lee, Changsop Yang, Chan-Young Kwon, Jongchul Keum, Jung-Hee Jang

**Affiliations:** ^1^KM Science Research Division, Korea Institute of Oriental Medicine, Daejeon, Republic of Korea; ^2^Hwamok Korean Medicine Clinic, Busan, Republic of Korea; ^3^Busan Association of Korean Medicine, Busan, Republic of Korea; ^4^Department of Oriental Neuropsychiatry, College of Korean Medicine, Dong-Eui University, Busan, Republic of Korea; ^5^Kukjeon Kyunghee Korean Medicine Clinic, Busan, Republic of Korea

**Keywords:** acupuncture, cognitive dysfunction, community-based participatory research, herbal medicine, near-infrared spectroscopy

## Abstract

**Background:**

Mild cognitive impairment (MCI) is a growing concern among older adults, with limited effective pharmacological treatments available. Despite the potential of herbal medicine and acupuncture in managing MCI, there is a lack of research on their long-term effects on cognitive function and brain activity in clinical practice settings. This study aimed to address this gap by exploring the effects of a community-based program integrating herbal medicine and acupuncture on cognitive function and neural responses in older individuals with MCI.

**Methods:**

Nineteen individuals were enrolled from a pool of 250 individuals registered in the 2021 Busan Dementia Prevention & Care Program. Participants with MCI received herbal medicine, acupuncture, and pharmacopuncture treatments over a 6-month period. The Montreal Cognitive Assessment (MoCA) was administered at baseline and after 3 and 6 months to evaluate cognitive function. Functional near-infrared spectroscopy (fNIRS) was used to measure prefrontal cortex activity during cognitive task performance, including verbal fluency, Stroop color and word, and digit span backward tests.

**Results:**

Seventeen participants (13 female; mean age, 69.5 years) with MCI completed the study. Following the 6-month intervention, they exhibited a significant increase in the MoCA total score over time [F_(2.32)_ =10.59, *p* < 0.0001]. Additionally, the deoxygenated hemoglobin beta coefficient in the left frontopolar prefrontal cortex significantly decreased during the Stroop task after the intervention.

**Conclusion:**

The Dementia Prevention & Care Program, which integrates herbal medicine and acupuncture, may enhance cognitive function in individuals with MCI. Moreover, the observed changes in prefrontal cortex activity after completion of the program suggest a need for further investigation of the underlying mechanisms.

## 1 Introduction

Mild cognitive impairment (MCI) is characterized by complaints regarding cognitive function accompanied by an objectively observed cognitive decline that exceeds what is considered normal for one's age. Unlike those with dementia, individuals with MCI typically maintain their daily functioning (1). The prevalence of MCI rises with age, estimated at 6.7%−8.4% in individuals aged 60 years and older and increasing to 25.2% among those older than 80 years (2). For individuals with MCI aged 70 years and older, the annual progression rate to dementia is approximately 5%−6% (3). Despite its prevalence and potential progression to dementia, there is currently no consensus on effective pharmacological treatments for MCI (2).

Emerging research suggests that herbal medicine and acupuncture may hold promise in slowing the progression of MCI and enhancing cognitive function (4, 5). In Korea, these interventions are being incorporated into community-based strategies for dementia prevention and management. Since 2016, Busan has implemented the Korean Medicine Dementia Prevention & Care Program led by Busan Metropolitan City, the Busan Association of Korean Medicine, and 16 dementia care centers. This program provides complimentary herbal medicine and pharmacopuncture treatments for individuals aged 60 years and older who reside in Busan, with acupuncture treatment costs covered by the participants themselves (6). Previous studies have reported improvements in cognitive function following these programs, along with high participant satisfaction and willingness to re-engage (6, 7).

The potential mechanisms underlying these improvements have been explored using various neuroimaging techniques. Studies utilizing functional magnetic resonance imaging (fMRI) have shown that herbal medicine treatments for patients with MCI can lead to decreased activation in the dorsolateral prefrontal cortex (DLPFC) and parietal cortex and increased activation in medial prefrontal, parietal, and temporal cortices (8). In addition, acupuncture treatment have been found to modulate prefrontal activation in patients with MCI (9).

Another method to investigate these mechanisms is functional near-infrared spectroscopy (fNIRS) (10). fNIRS, a non-invasive optical method, utilizes near-infrared light to monitor changes in cerebral blood hemodynamics associated with neural activity by monitoring the levels of oxy and deoxygenated hemoglobin (HbO_2_ and HbR, respectively), which serve as indicators of changes in regional cerebral blood flow (11). Compared to other functional imaging techniques, fNIRS offers advantages such as portability, relative insensitivity to movement, and absence of the need for injection of radioactive compounds (12).

fNIRS is a suitable tool for evaluating hemodynamic changes during the performance of cognitive tasks in patients with MCI (13). Studies utilizing fNIRS have reported that cerebral blood flow is reduced in individuals with MCI and Alzheimer's disease (AD) compared with that in healthy aging, as demonstrated in single-photon emission computed tomography, positron emission tomography, and functional magnetic resonance imaging studies (14). Consistent with this, patients with MCI and AD have also shown reduced frontal oxygenation across various cognitive domains, including tasks involving word retrieval, memory, motor control, and visuospatial perception, as observed in fNIRS studies (13–15). In a study evaluating acupuncture treatment using fNIRS, the prefrontal activation was modulated after acupuncture treatment during 12 weeks and exhibited a strong HbO_2_ response, which is significantly similar to those of the healthy control's averaged response (16). However, there is a lack of research measuring brain activity in patients with MCI in the context of long-term herbal medicine and acupuncture treatments used in clinical practice setting.

This study aimed to address the gap in long-term, clinical practice-based research by investigating the effects of combined herbal medicine and acupuncture treatment on cognitive function and cerebral hemodynamic changes in patients with MCI. Specifically, we measured prefrontal cortex activity during cognitive task performance (verbal fluency, Stroop color and word, and digit span backward tests) using fNIRS before and after the 6-month intervention. By using fNIRS to measure prefrontal cortex activity during cognitive tasks before and after a 6-month intervention period, we aimed to provide insights into the neural mechanisms underlying potential cognitive improvements in patients with MCI.

## 2 Materials and methods

### 2.1 Study design

This retrospective observational study aimed to evaluate the effectiveness of Korean traditional medicine treatments in individuals with MCI and investigate the associated mechanisms, specifically focusing on prefrontal activation during cognitive tasks using fNIRS. Participants were recruited from among those registered in the 2021 Dementia Prevention & Care Program of the Busan Association of Korean Medicine. Written informed consent was obtained from all participants prior to registration. Participants registered in the 2021 Dementia Prevention & Care Program received a combination of Korean medicine interventions, including herbal medicine, acupuncture, and pharmacopuncture, over a 6-month period. The outcome assessment included the measurement of cognitive function; hemodynamic changes in the prefrontal cortex (PFC) during cognitive tasks were measured in participants who enrolled in this study. Individuals without cognitive decline, who served as healthy controls, were also enrolled. The study was conducted at two Korean medicine clinics in Busan, with enrollment occurring from January 13, 2021 to April 21, 2021, and a final follow-up on November 23, 2021. Ethical approval was obtained from the Institutional Review Board of the Korea Institute of Oriental Medicine (I-2307/007-001).

### 2.2 Participants

Nineteen patients with MCI were enrolled in two Korean medicine clinics. Participants of the 2021 Dementia Prevention & Care Program in Busan who expressed a willingness to undergo additional assessment using fNIRS were included in this study. The inclusion criteria for the 2021 Dementia Prevention & Care Program in Busan were as follows: participants who were over 60 years old; diagnosed with MCI based on cognitive assessments such as the Korea Dementia Screening Questionnaire (17), Cognitive Impairment Screening Test (CIST) (18), and Montreal Cognitive Assessment (MoCA) (19, 20) with a score below 23; willingness to receive herbal medicine, acupuncture, and pharmacopuncture treatment; able to visit the clinic twice a week; and provided voluntary informed consent. The exclusion criteria included current use of anti-dementia drugs, history of severe mental disorders, unresolved cancer diagnosis, and ineligibility for enrollment for other reasons.

Nine healthy controls with normal cognitive functions were enrolled in this study. The healthy controls were recruited from the same Korean medicine clinics. Participants without complaints of cognitive decline underwent MoCA screening, and those with MoCA scores > 22 were included as healthy controls.

### 2.3 Intervention

Participants with MCI underwent a 6-month treatment as part of the Dementia Prevention & Care Program in Busan, which included herbal medicine, acupuncture, and pharmacopuncture. The herbal medicine consisted of three individualized prescriptions: Kami Guibi-tang (17, 18), Yukmijihwang-tang (19), and Dangguijagyag-san (20, 21). These prescriptions were determined using the Korean medicine pattern identification system, which considers factors such as appetite, sleep, stool frequency and form, urine, sensitivity to fever or cold, exercise habits, and stress levels. The three prescriptions were finalized after application and modification processes in the Dementia Prevention & Care Program in Busan, which began in 2016. Initially, there were six patterns and corresponding prescriptions, including Bojungikgi-tang for qi deficiency, Palmijihwang-tang for yang deficiency, and Gyejibokryeong-hwan for blood stasis. However, Bojungikgi-tang and Palmijihwang-tang were often unsuitable for long-term use, and there were few patients with the blood stasis pattern. Consequently, the program was refined to three patterns and their corresponding prescriptions. Kami Guibi-tang was prescribed to patients with qi and blood deficiency patterns, Yukmijihwang-tang for yin deficiency patterns, and Dangguijagyag-san for blood deficiency patterns. Korean medicine doctors confirmed the appropriateness of the pattern identification and prescription based on each patient's condition. The participants took the herbal medicines twice daily for 6 months. The herbs in the three herbal medicine granules are listed in Table 1. The herbal medicine granules were manufactured by Kracie Pharma, Ltd. in accordance with Good Manufacturing Practice under the guidelines of the Ministry of Food and Drug Safety.

**Table 1:** Names and dosages of herbal ingredients for the three herbal formulas used in this study.

**Formula**	**Herbal name**	**Source species**	**Part used**	**Dosage**
Kami Guibi-tang (7.5 g/day)	Ginseng Radix	*Panax ginseng* C.A. Mey.	Root	3.0 g
	Atractylodis Rhizoma Alba	*Atractylodes macrocephala* Koidz.	Rhizome	3.0 g
	Poria Sclerotium	*Poria cocos* Wolf	Sclerotium	3.0 g
	Zizyphi Semen	*Ziziphus jujuba* Mill.	Seed	3.0 g
	Longan Arillus	*Dimocarpus longan* Lour.	Arill	3.0 g
	Astragali Radix	*Astragalus mongholicus* Bunge	Root	2.0 g
	Angelicae Gigantis Radix	*Angelica gigas* Nakai	Root	2.0 g
	Polygalae Radix	*Polygala senega* L.	Root	1.5 g
	Bupleuri Radix	*Bupleurum chinense* DC.	Root	3.0 g
	Gardeniae Fructus	*Gardenia jasminoides* J. Ellis	Fruit	2.0 g
	Glycyrrhizae Radix et Rhizoma	*Glycyrrhiza glabra* L.	Root and rhizome	1.0 g
	Aucklandiae Radix	*Dolomiaea costus* (Falc.) Kasana & A.K. Pandey	Root	1.0 g
	Zizyphi Fructus	*Ziziphus jujuba* Mill.	Fruit	1.5 g
	Zingiberis Rhizoma	*Zingiber officinale* Roscoe	Rhizome	0.5 g
Yukmijihwang-tang (6.0 g/day)	Rehmanniae Radix Preparata	*Rehmannia glutinosa* (Gaertn.) DC.	Root	5.0 g
	Dioscoreae Rhizoma	*Dioscorea polystachya* Turcz.	Rhizome	3.0 g
	Corni Fructus	*Cornus officinalis* Siebold & Zucc.	Fruit	3.0 g
	Poria Sclerotium	*Poria cocos* Wolf	Sclerotium	3.0 g
	Moutan Radicis Cortex	*Paeonia suffruticosa* Andrews	Rhizodermis	3.0 g
	Alismatis Rhizoma	*Alisma plantago-aquatica* subsp. *orientale* (Sam.)	Rhizome	3.0 g
Dangguijagyag-san (6.0 g/day)	Angelicae Gigantis Radix	*Angelica gigas* Nakai	Root	3.0 g
	Cnidii Rhizoma	*Ligusticum officinale* (Makino) Kitag.	Rhizome	3.0 g
	Paeoniae Radix	*Paeonia lactiflora* Pall.	Root	6.0 g
	Poria Sclerotium	*Poria cocos* Wolf	Sclerotium	4.0 g
	Atractylodis Rhizoma Alba	*Atractylodes lancea* (Thunb.) DC.	Rhizome	4.0 g
	Alismatis Rhizoma	*Alisma plantago-aquatica* subsp. *orientale* (Sam.)	Rhizome	3.0 g

The participants also received acupuncture and pharmacopuncture treatments twice a week for 6 months. The acupuncture involved points on the head (EX-HN1), upper body (bilateral PC6, HT7, and PC8), and lower body (bilateral ST36). Stainless steel acupuncture needles (0.25 × 30 mm; SMC Co., Korea) were used, and each acupuncture session lasted 20 minutes. Pharmacopuncture was administered at six acupoints: GV16, GV14, and bilateral GB20 and GB21. A dosage of 0.1–0.2 cc of Hominis Placenta pharmacopuncture (22) was injected. The participants enrolled as healthy controls did not undergo any interventions for cognitive function.

### 2.4 Measurements

Demographic information (sex, age, education level, and occupational status), comorbid diseases, and MoCA scores (23, 24) were collected from both groups of participants. As a cognitive assessment tool, the MoCA was administered three times to participants with MCI (at baseline, after 3 months, and after 6 months) to track changes in cognitive function. For healthy controls, the MoCA was administered once at baseline to confirm their cognitive status. The MoCA evaluates seven cognitive domains, namely executive/visuospatial function, naming, attention, language, abstraction, recall, and orientation, with a maximum score of 30 (23, 24). A previous study suggested a cutoff score of 22/23 for screening MCI in older Korean outpatients (24).

In patients with MCI, the CIST (25) and Geriatric Depression Scale (GDepS) short form (26, 27) were administered twice, at baseline and after 6 months of treatment. The CIST was developed by the Ministry of Health and Welfare in Korea and consists of 13 items covering six cognitive domains (orientation, attention, visuospatial function, executive function, memory, and language). The scores range from 0 to 30, with higher scores indicating better cognitive function (25). GDepS assesses the severity of geriatric depression, with scores ranging from 0 to 15. A higher score indicated more severe depression (26). Additionally, the occurrence of adverse events was meticulously recorded. The participants were instructed to report any instances of nausea, vomiting, decreased appetite, diarrhea, constipation, headache, dizziness, abdominal pain, chest pain, fatigue, insomnia, swelling, weight loss, drowsiness, or other relevant symptoms.

### 2.5 fNIRS and cognitive tasks

**Figure 1:**
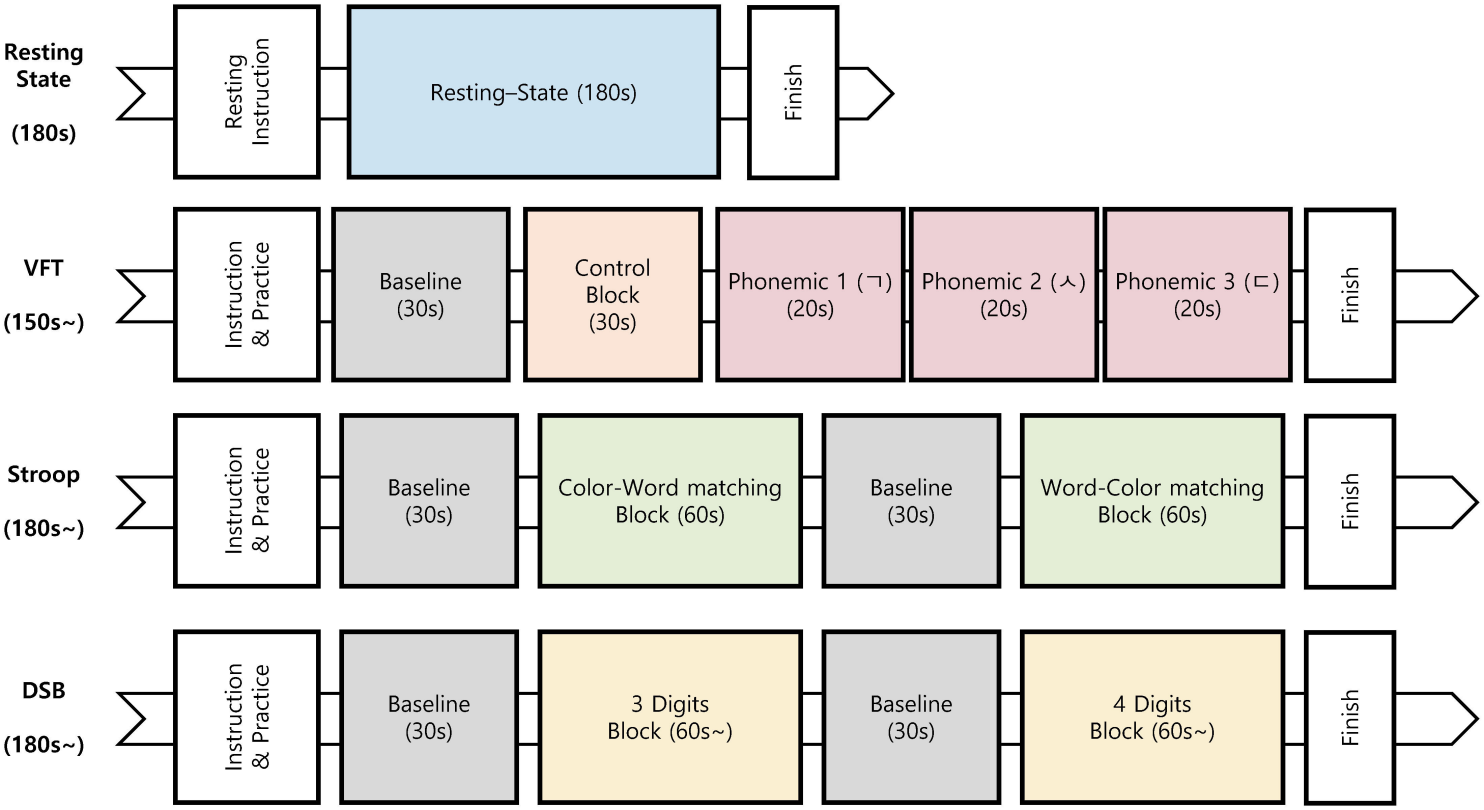
Procedure for the resting state and cognitive tasks. VFT, verbal fluency test; Stroop, Stroop color and word test; DSB, digit span backward test.

PFC activity was measured using a portable fNIRS device (Supplementary Figure 1) during both the resting state and three consecutive cognitive tasks (Figure 1). Measurements were recorded three times in patients with MCI (at baseline, after 3 months, and after 6 months) and twice in healthy controls (at baseline and after 6 months).

#### 2.5.1 Cognitive tasks

**Figure 2:**
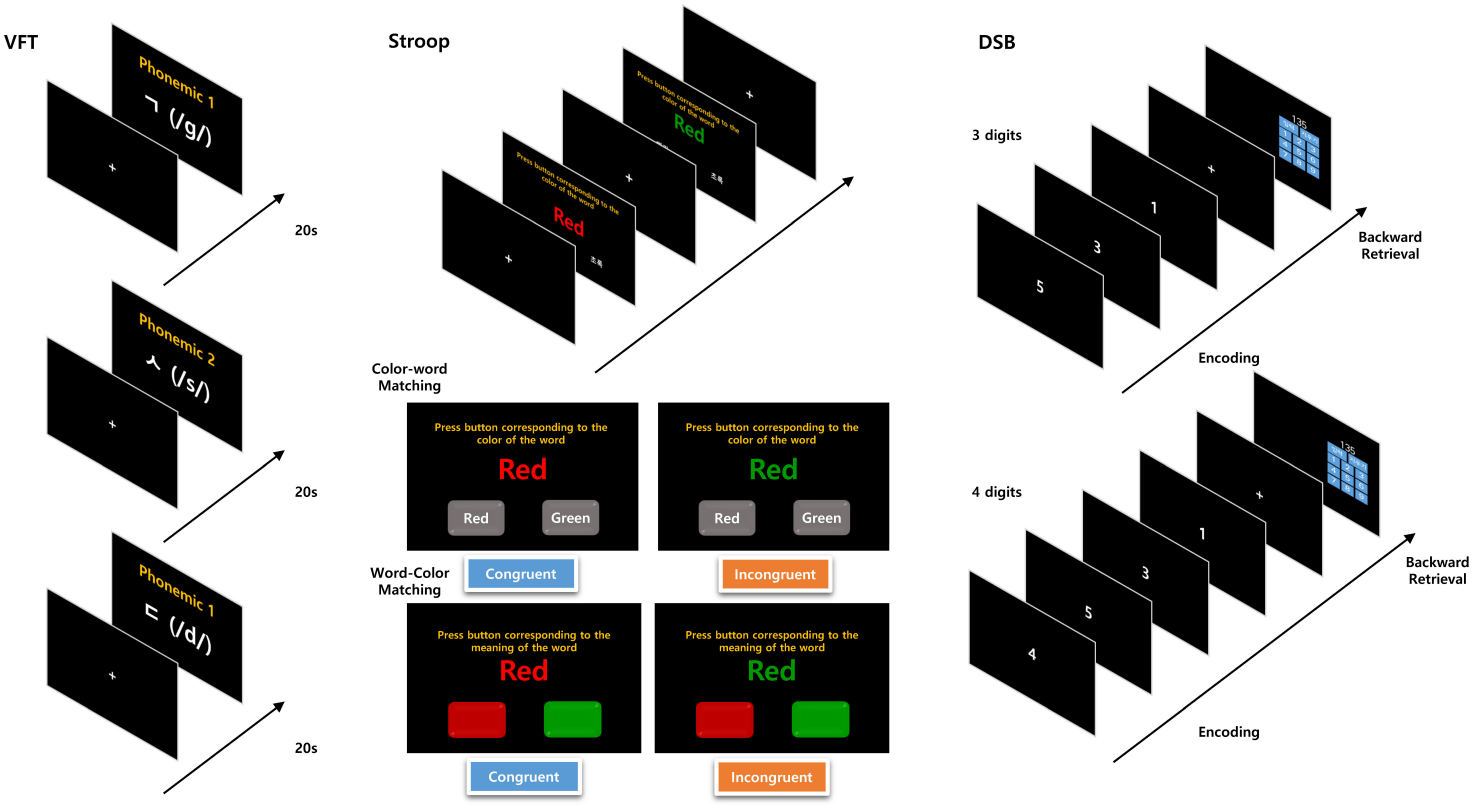
Protocols for VFT, Stroop color and word test, and DSB test.

The cognitive tasks included the verbal fluency test (VFT), Stroop color and word test (Stroop), and digit span backward test (DSB). Each cognitive task was comprised of low-difficulty (easy) and high-difficulty (hard) tasks. Cognitive tasks were administered via a touch monitor, and the software for these tasks was developed using a Python-based psychological experiment toolbox (Figure 2) (28).

In the VFT, participants are required to retrieve and articulate words corresponding to given cues within a limited timeframe. For the high-difficulty component, a phonemic VFT (29) was conducted, in which participants were asked to spontaneously report words beginning with each phonemic (three Korean consonant letters: /g/, /s/, and /d/) within 20 s for each letter. For the low-difficulty component, the participants were instructed to simply articulate vowels.

The Stroop test includes color–word matching (Stroop) and word–color matching (reverse Stroop) (30). There were 24 questions delivered in two sessions, with each question presenting two color names (red and green) displayed in matched or unmatched color ink on a black background. During the color–word matching phase (low-difficulty task; 60 s duration), participants were required to select the word button corresponding to the color of the word. Conversely, during the word–color matching phase (high-difficulty task), which also lasted for 60 s, the participants were instructed to select the color button corresponding to the meaning of the word.

The DSB test requires participants to memorize a series of numbers and then recall them in reverse order (31). The test included five tasks of memorizing three consecutive numbers (low-difficulty task) and four tasks of memorizing four consecutive numbers (high-difficulty task). The participants were instructed to enter the numbers within 7 s for each set of three consecutive numbers, and within 10 s for each set of four consecutive numbers.

#### 2.5.2 fNIRS data acquisition and preprocessing

**Figure 3:**
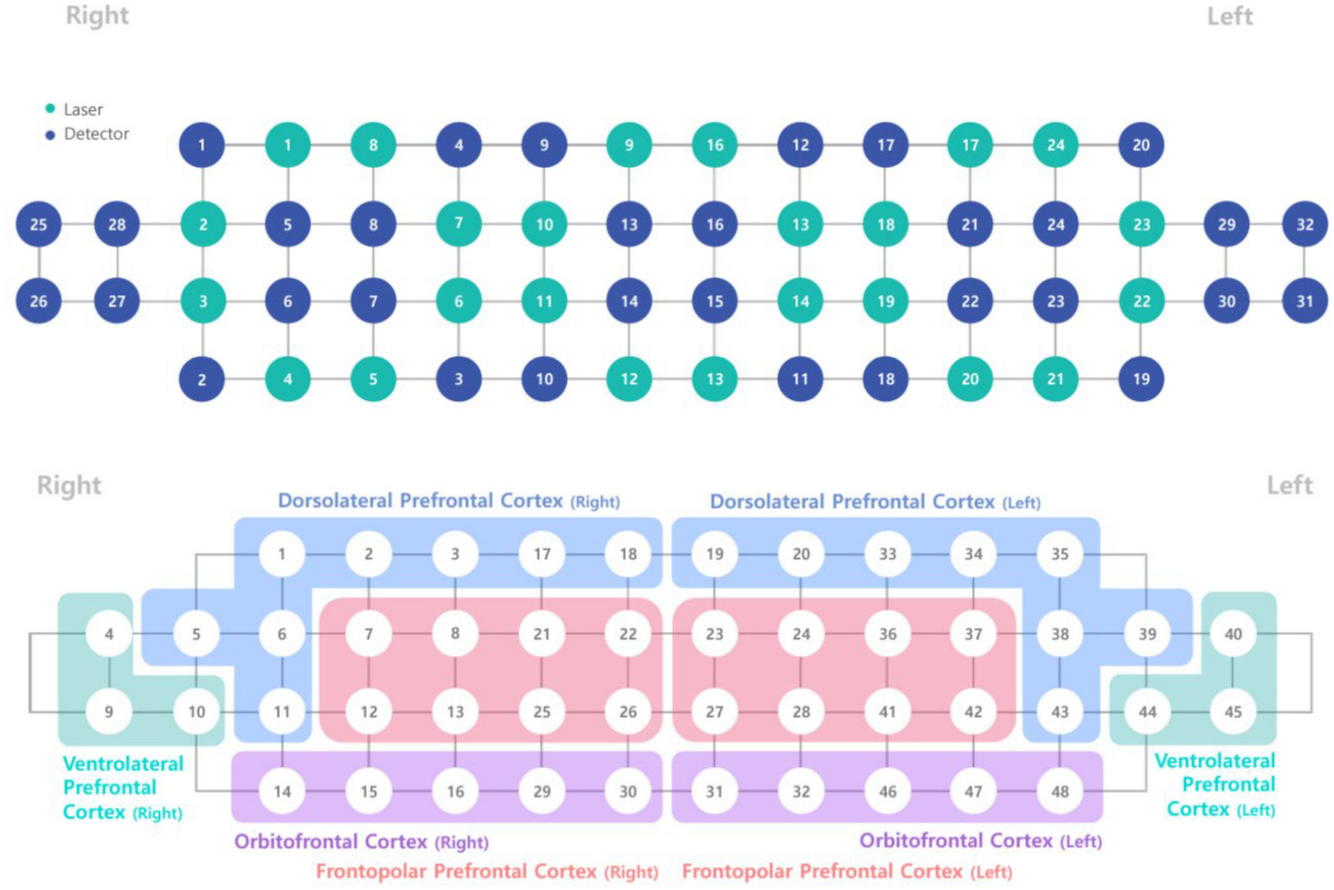
Arrangement of sources and detectors and configuration of region-of-interest channels.

The fNIRS device (NIRSIT; OBELAB, Inc., Seoul, Korea) used in this study consisted of a standard configuration featuring 48 channels with a source-detector distance of 3 cm (Figure 3). It is designed to measure hemodynamic responses in the prefrontal brain regions, including the right and left dorsolateral prefrontal cortex (DLPFC; eight channels each), ventrolateral prefrontal cortex (VLPFC; three channels each), orbitofrontal cortex (OFC; five channels each), and frontopolar cortex (FPC; eight channels each). Near-infrared light beams at two wavelengths (780 and 850 nm) were emitted into these regions to assess variations in cerebral blood oxygen saturation by measuring HbO_2_ and HbR, respectively (32).

fNIRS data preprocessing and analysis were performed using MATLAB (version 2023b, MathWorks, United States). Data from 48 channels with a 3 cm source-detector distance were processed. Prior to analysis, task data were isolated to eliminate unnecessary time windows between tasks (33, 34). The preprocessing pipeline began with signal quality control. Negative intensity values due to saturation were replaced with neighboring non-negative values. Channels were then rejected based on established criteria (35, 36): median intensity below 20 A.U. (above which the observed cardiac pulsation becomes prominent), coefficient of variation exceeding 7.5% (36), consecutive values indicating saturation for more than 5% of the time series, extremely negative correlation (< -0.9) between HbO and HbR (35). The rejected channel values were substituted with the averages of the neighboring channels in the same Brodmann region. Next, data conversion and artifact correction were performed. Intensity data were converted to delta optical density (dOD) using the mean intensity of each channel wavelength as baseline (36). Detrending was applied to remove slow drift from dOD data. Motion artifact were then corrected using temporal derivative distribution repair (TDDR) algorithm (37). For concentration calculation, based on modified Beer-Lambert law (38, 39), dOD data were converted to Hb concentration changes (mM.mm) with molar extinction coefficients (40). Lastly, a 5^th^ order Butterworth bandpass filter was applied, with a low pass cutoff frequency of 0.1 Hz and a high pass cutoff frequency of 0.01 Hz to remove potential physiological noise such as cardiac signal and measurement drift.

#### 2.5.3 fNIRS signal analysis

fNIRS signal analysis was conducted using the general linear model (GLM) approach, employing beta estimation with the ordinary least squares solution. Task design regressors were constructed for each task and convolved with hemodynamic response functions to model task-induced HbO_2_ and HbR brain activation signals. A high HbO_2_ beta value indicated increased HbO_2_ concentration changes (indicating activation) by the task, whereas a high HbR beta value indicated decreased HbR concentration changes (also, indicating activation) by the task. The main regressors for each cognitive task included the high-difficulty task (hard), low-difficulty task (easy), and resting state. Contrasts for regressors of interest were established to compare hard and easy tasks, with the aim of identifying channels showing a differential response between the two task conditions. HbO_2_ and HbR beta coefficient in each channel were estimated using the GLM, and channel estimates were averaged to yield summary estimates by Brodmann regions, including the right and left DLPFC, VLFPC, OFC, and FPC.

### 2.6 Statistical analysis

The changes in variables over time within the MCI patient group were initially examined. For datasets with three time points (baseline, after 3 months, after 6 months), analysis was performed using Repeated Measures ANOVA or Friedman Rank Sum Test. Pairwise comparisons were conducted using paired *t-*tests or paired Wilcoxon tests, with Holm correction for multiple comparison. Results at 3 months and 6 months were compared to baseline, respectively. For datasets with two time points (baseline, after 6 months), paired *t-*tests or paired Wilcoxon tests were applied. Between-group comparisons (MCI patients vs. Healthy controls) were conducted by comparing the changes within each group (values at 6 months minus baseline). The Mann-Whitney U test was used to compare the changes from baseline in both groups. All statistical analyses were carried out using R 4.3.1 [R Core Team, (52)].

## 3 Results

### 3.1 Demographic and participant characteristics

**Figure 4:**
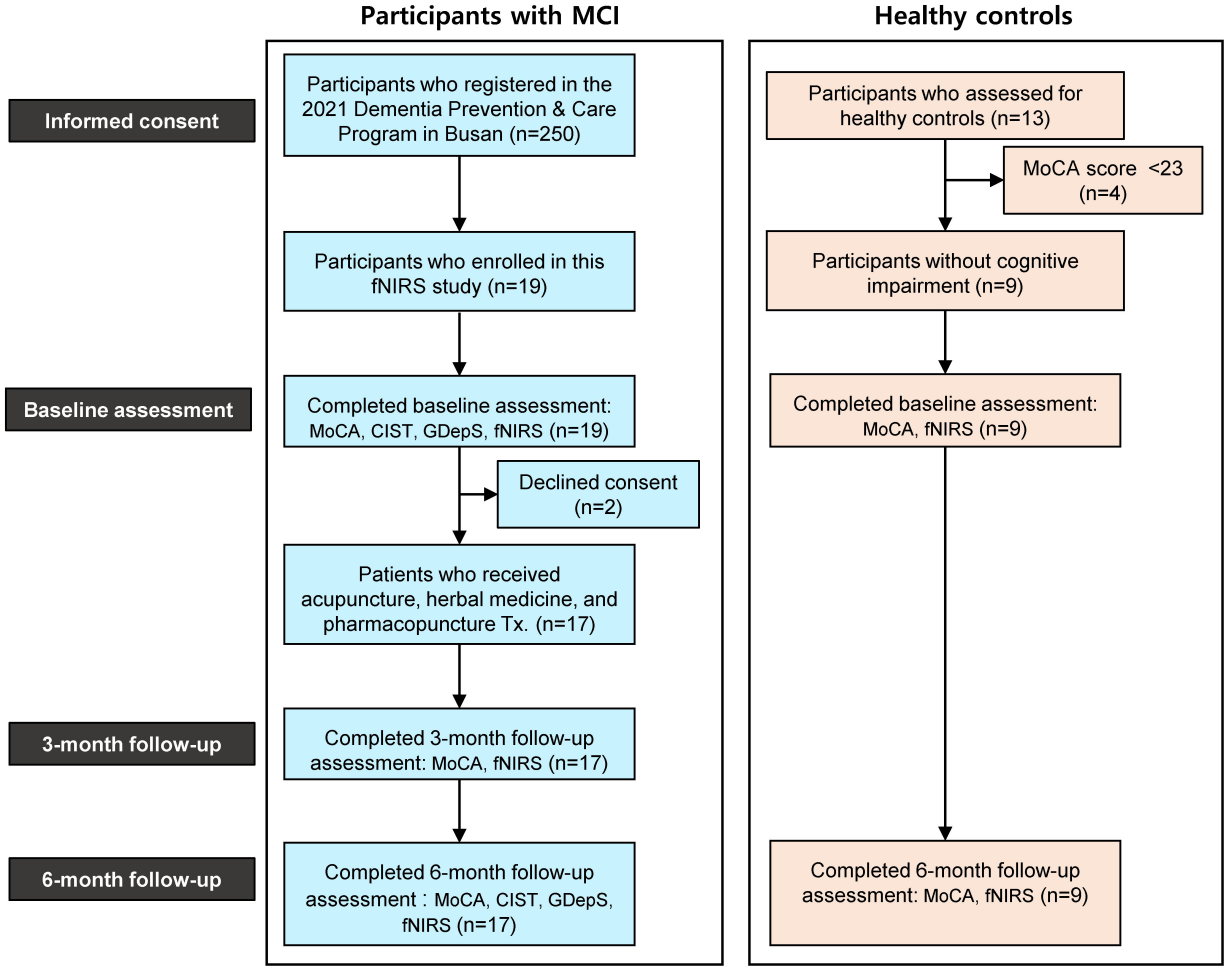
Study flow diagram.

A total of 19 participants with MCI were enrolled in this study. Out of the 19 participants with MCI, 2 participants dropped out due to declined consent, and 1 participant lacked fNIRS data at the 3-month follow up. The analysis for fNIRS data included the remaining 16 participants with complete data. Separately, for the healthy controls with normal cognitive function, 13 participants primarily with musculoskeletal diseases and without cognitive decline complaints were assessed for eligibility. Four participants with a MoCA score under 23 were excluded, and 9 participants were enrolled as healthy controls (Figure 4).

The demographic and baseline characteristics of the study participants are detailed in Table 2. The distribution of age, sex, education levels, and occupation demonstrated comparability between the Mild Cognitive Impairment (MCI) and healthy control groups, with 76.5% women in the MCI group and 66.7% in the healthy control group. However, the prevalence of comorbidities varied significantly between the two groups. Meanwhile, the baseline MoCA scores were markedly lower in the MCI group (19.9 ± 1.2) compared to the healthy control group (26.0 ± 2.5).

**Table 2:** Demographic and baseline characteristics of the study participants.

	**Participants with MCI (*n =* 17)**	**Healthy controls (*n =* 9)**	***p*-value**
**Age**	69.5 ± 4.4	71.4 ± 3.8	0.268
**Sex**			
Men	4 (23.5)	3 (33.3)	0.943
Women	13 (76.5)	6 (66.7)	
**Education level**			
≤ 6 years	10 (58.8)	4 (44.4)	0.775
>6 years	7 (41.2)	5 (55.6)	
**Occupation**			
Employed	11 (64.7)	2 (22.2)	0.099
Unemployed	6 (35.3)	7 (77.8)	
**Comorbidities**			
Hypertension	8 (47.1)	0 (0.0)	0.043
Hyperlipidemia	6 (35.3)	0 (0.0)	0.123
Diabetes	3 (17.6)	0 (0.0)	0.487
Musculoskeletal diseases	4 (23.5)	8 (88.9)	0.006
Insomnia	1 (5.9)	1 (11.1)	0.999
**MoCA score (baseline)**	19.9 ± 1.2	26.0 ± 2.5	< 0.001

Continuous variables are presented as the mean ± standard deviation and categorical variables are expressed as numbers (percentages). MCI, mild cognitive impairment; MoCA, Montreal Cognitive Assessment.

### 3.2 Changes in MoCA, CIST, and GDepS score in participants with MCI

Table 3 presents the changes in MoCA, CIST, and GDepS score in participants with MCI over 6-month period. Following the herbal medicine, acupuncture, and pharmacopuncture treatment, the MoCA total score in participants with MCI exhibited a notable increase, rising by 3.24 points (95% CI, 1.87 to 4.60) compared to baseline after 6 months. Concurrently, the mean CIST score also increased, indicative of cognitive improvement, while the mean GDepS score decreased, suggesting a decline in depression symptoms. Additionally, one case of headache was reported as an adverse event following pharmacopuncture treatment. The severity of the headache was mild, and it resolved spontaneously. No other adverse events were reported.

**Table 3:** Changes in the MoCA, CIST, and GDepS scores in participants with MCI (*n* = 17).

	**Baseline**		**3 months**		**6 months**		**Repeated measures ANOVA**	**Pairwise comparison**	
	**Mean**	**SD**	**Mean**	**SD**	**Mean**	**SD**		**Baseline vs. 3 months**	**Baseline vs. 6 months**
MoCA	19.9	1.2	21.4	2.8	23.1	2.9	F_(2, 32)_ = 10.59, *p < * 0.0001	1.47, *p =* 0.1080	3.24, *p =* 0.0002
CIST	22.9	3.2	-	-	25.0	2.8	-	-	2.12, *p =* 0.006
GDepS	3.2	2.6	-	-	1.8	2.1	-	-	−1.41, *p =* 0.019

MoCA, CIST, and GDepS scores were measured in 17 participants with MCI, and the fNIRS measurement was omitted in one of them. MoCA, montreal cognitive assessment; CIST, Cognitive Impairment Screening Test; GDepS, Geriatric Depression Scale (short form); SD, standard deviation.

### 3.3 Changes in HbO_2_ and HbR concentration during cognitive tasks

**Figure 5:**
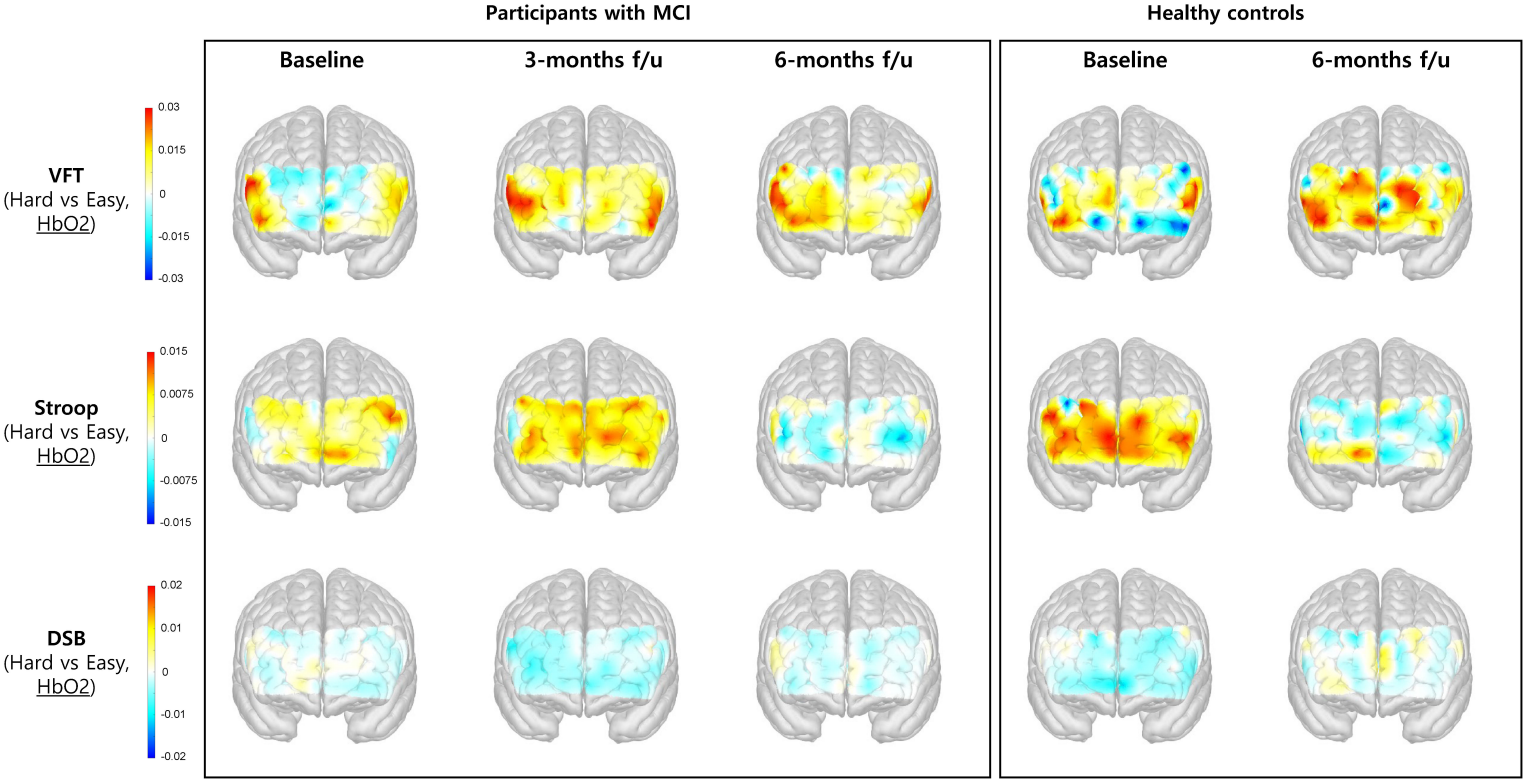
Changes in the concentration of HbO_2_ in the prefrontal cortex during performance of cognitive tasks, comparing hard vs. easy tasks. The color bar indicates the beta coefficient of each hemoglobin concentration change, where the warmer colors indicate activation and the cooler colors indicate deactivation. Note that the warmer colors in the HbO_2_ beta amplitude map indicate activation in the region, meaning that the overall concentration change of HbO_2_ is increased. f/u, follow up.

**Figure 6:**
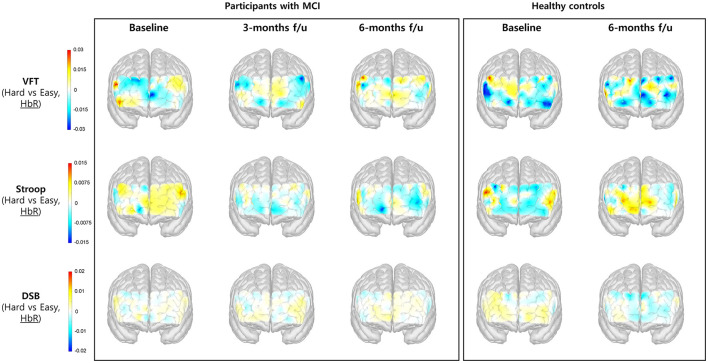
Changes in the concentration of HbR in the prefrontal cortex during performance of cognitive tasks, comparing hard vs. easy tasks. The color bar indicates the beta coefficient of each hemoglobin concentration change, where the warmer colors indicate activation and the cooler colors indicate deactivation. Note that the warmer colors in the HbR beta amplitude map indicate activation in the region, meaning that the overall concentration change of HbR is decreased.

Table 4 presents the observed changes in cognitive tasks (VFT, Stroop, and DSB) among patients with MCI. While a general trend toward improvement over time was noted, many of these changes did not attain statistical significance. Throughout the administration of the cognitive tasks, HbO_2_ and HbR signals were measured in each channel, and the differences in signal between the high- and low-difficulty cognitive tasks were computed. The HbO_2_ and HbR signals in PFC among participants with MCI and healthy controls over time are illustrated in Figures 5, 6, respectively. The color bar on the left side of each figure indicates normalized signal beta amplitude. Additionally, the HbO_2_ and HbR beta values for each prefrontal region at baseline and 6-month follow-up are presented in Tables 5, 6, respectively.

**Table 4:** Changes in cognitive task scores in participants with MCI (*n* = 16).

	**Baseline**		**3 months**		**6 months**		**Friedman test**	**Pairwise comparison**	
	**Median**	**IQR**	**Median**	**IQR**	**Median**	**IQR**		**Baseline vs. 3 months**	**Baseline vs. 6 months**
VFT	9.0	7.0	6.0	6.2	10.5	5.5	$\chi_{\left(2\right)}^{2}$ = 13.9, *p =* 0.0010	−1.00, *p =* 0.3630	4.00, *p =* 0.0970
Stroop: C-W	64.6	43.8	77.1	46.9	87.5	18.8	$\chi_{\left(2\right)}^{2}$ = 7.3, *p =* 0.0258	−4.17, *p =* 0.7060	16.67, *p =* 0.1160
Stroop: W-C	52.1	55.2	70.8	35.4	64.6	41.7	$\chi_{\left(2\right)}^{2}$ = 1.9, *p =* 0.3867	12.50, *p =* 0.6520	0.00, *p =* 0.7330
DSB: 3 digits	40.0	40.0	50.0	45.0	80.0	40.0	$\chi_{\left(2\right)}^{2}$ = 6.4, *p =* 0.0414	20.00, *p =* 0.1070	20.00, *p =* 0.0310
DSB: 4 digits	12.5	25.0	20.0	25.0	25.0	25.0	$\chi_{\left(2\right)}^{2}$ = 4.04, *p =* 0.1324	0.00, *p =* 0.9200	25.00, *p =* 0.1860

IQR, interquartile range; VFT, verbal fluency test; C, color; W, word; DSB, digit span backward test. The percentage of correct answers (%) was calculated for the Stroop and DSB tasks.

**Table 5:** Changes in the beta value of oxygenated hemoglobin (HbO_2_) in the prefrontal cortex during cognitive tasks, comparing hard vs. easy tasks.

	**Participants with MCI**				**Within-group comparison (in patients with MCI)**		**HC**				**Between-group comparison (**Δ **within MCI vs**. Δ **within HC)**	
	**Baseline**		**6 months**				**Baseline**		**6 months**		**W**_Mann − Whitney_	***p*** **value**
	**Median**	**IQR**	**Median**	**IQR**	**Estimate**	***p*** **value**	**Median**	**IQR**	**Median**	**IQR**		
**VFT**												
DLPFC_L	0.0097	0.0305	0.0031	0.0270	0.0043	0.678	−0.0070	0.0473	0.0010	0.0447	66.00	0.597
DLPFC_R	−0.0015	0.0319	0.0091	0.0278	0.0099	0.404	0.0057	0.0244	0.0161	0.0268	84.00	0.711
VLPFC_L	0.0158	0.0394	0.0289	0.0248	0.0164	0.348	0.0324	0.0657	0.0114	0.0426	44.00	0.238
VLPFC_R	0.0367	0.0602	0.0111	0.0509	−0.0109	0.632	0.0195	0.0781	0.0229	0.1290	76.00	0.846
OFC_L	0.0062	0.0327	0.0163	0.0160	0.0064	0.487	0.0182	0.0578	0.0153	0.0491	88.00	0.560
OFC_R	0.0078	0.0417	0.0161	0.0379	0.0064	0.459	0.0220	0.0357	0.0274	0.0326	81.00	0.833
FPC_L	−0.0001	0.0353	0.0008	0.0121	0.0053	0.517	0.0149	0.0348	0.0239	0.0290	83.00	0.751
FPC_R	−0.0017	0.0193	0.0012	0.0248	0.0126	0.064	0.0299	0.0283	0.0176	0.0248	50.00	0.164
**Stroop**												
DLPFC_L	0.0034	0.0100	0.0007	0.0115	−0.0026	0.495	0.0082	0.0133	−0.0004	0.0075	62.00	0.598
DLPFC_R	−0.0002	0.0108	−0.0004	0.0101	−0.0030	0.284	0.0078	0.0123	0.0016	0.0105	54.00	0.241
VLPFC_L	0.0008	0.0223	−0.0002	0.0108	0.0020	0.744	0.0014	0.0192	0.0009	0.0038	57.00	0.697
VLPFC_R	−0.0012	0.0132	0.0038	0.0279	0.0054	0.433	0.0015	0.0113	−0.0038	0.0179	43.00	0.108
OFC_L	0.0038	0.0109	−0.0002	0.0082	−0.0022	0.487	0.0118	0.0089	−0.0025	0.0170	52.00	0.200
OFC_R	0.0029	0.0238	0.0026	0.0109	−0.0037	0.159	0.0100	0.0129	0.0090	0.0098	79.00	0.916
FPC_L	0.0032	0.0202	−0.0013	0.0145	−0.0036	0.597	0.0098	0.0151	0.0002	0.0109	53.00	0.220
FPC_R	0.0029	0.0143	0.0001	0.0152	−0.0029	0.329	0.0107	0.0100	0.0000	0.0084	42.00	0.066
**DSB**			
DLPFC_L	−0.0010	0.0049	−0.0004	0.0035	0.0005	0.747	−0.0019	0.0028	−0.0014	0.0030	90.00	0.491
DLPFC_R	−0.0008	0.0040	0.0004	0.0059	0.0005	0.890	−0.0013	0.0063	−0.0015	0.0091	84.00	0.711
VLPFC_L	−0.0032	0.0073	−0.0006	0.0132	0.0019	0.404	−0.0035	0.0083	0.0016	0.0074	75.00	0.528
VLPFC_R	0.0003	0.0161	0.0009	0.0109	0.0053	0.263	−0.0010	0.0048	−0.0013	0.0059	61.00	0.711
OFC_L	−0.0014	0.0126	0.0003	0.0089	0.0000	1.000	−0.0043	0.0040	−0.0017	0.0058	85.00	0.487
OFC_R	−0.0006	0.0115	−0.0020	0.0095	−0.0018	0.487	−0.0050	0.0050	0.0009	0.0090	101.00	0.200
FPC_L	0.0010	0.0089	−0.0014	0.0059	−0.0012	0.353	−0.0046	0.0094	−0.0014	0.0051	109.00	0.085
FPC_R	0.0005	0.0077	−0.0006	0.0042	−0.0017	0.306	0.0011	0.0071	0.0021	0.0084	99.00	0.241

HC, healthy controls; L, left; R, right; DLPFC, dorsolateral prefrontal cortex; VLPFC, ventrolateral prefrontal cortex; FPC, frontopolar prefrontal cortex; OFC, orbitofrontal cortex.

**Table 6:** Changes in the beta value of deoxygenated hemoglobin (HbR) in the prefrontal cortex during cognitive tasks, comparing hard vs. easy tasks.

	**Participants with MCI**				**Within-group comparison (in patients with MCI)**		**HC**				**Between-group comparison (**Δ **within MCI vs**. Δ **within HC)**	
	**Baseline**		**6 months**				**Baseline**		**6 months**		**W**_Mann − Whitney_	***p*** **value**
	**Median**	**IQR**	**Median**	**IQR**	**Estimate**	***p*** **value**	**Median**	**IQR**	**Median**	**IQR**		
**VFT**												
DLPFC_L	0.0026	0.0084	0.0031	0.0110	0.0023	0.495	−0.0060	0.0225	−0.0168	0.0265	47.00	0.120
DLPFC_R	0.0033	0.0219	−0.0080	0.0322	0.0059	0.579	−0.0034	0.0230	0.0054	0.0388	79.00	0.916
VLPFC_L	−0.0025	0.0360	0.0081	0.0192	0.0211	0.117	−0.0017	0.0199	−0.0031	0.0246	45.00	0.264
VLPFC_R	0.0058	0.0492	0.0077	0.0443	0.0083	0.528	0.0020	0.0320	0.0248	0.0527	93.00	0.251
OFC_L	0.0006	0.0175	0.0041	0.0237	0.0014	0.678	−0.0024	0.0358	−0.0085	0.0402	76.00	0.999
OFC_R	0.0084	0.0184	−0.0045	0.0137	−0.0133	0.132	−0.0009	0.0216	−0.0014	0.0153	102.00	0.181
FPC_L	0.0004	0.0091	−0.0005	0.0057	0.0001	0.963	−0.0071	0.0151	−0.0088	0.0328	72.00	0.833
FPC_R	−0.0071	0.0123	0.0010	0.0180	0.0084	0.080	−0.0002	0.0132	−0.0065	0.0336	45.00	0.095
**Stroop**												
DLPFC_L	0.0017	0.0051	−0.0006	0.0049	−0.0024	0.034^*^	0.0006	0.0063	−0.0005	0.0036	79.00	0.718
DLPFC_R	−0.0001	0.0077	−0.0012	0.0042	−0.0018	0.431	0.0031	0.0145	0.0020	0.0133	62.00	0.458
VLPFC_L	−0.0026	0.0240	0.0019	0.0085	−0.0003	0.980	−0.0020	0.0065	0.0018	0.0045	74.00	0.569
VLPFC_R	−0.0007	0.0175	0.0041	0.0148	0.0061	0.252	−0.0002	0.0149	−0.0048	0.0088	48.00	0.187
OFC_L	0.0009	0.0030	−0.0013	0.0070	−0.0003	0.782	−0.0019	0.0101	0.0026	0.0063	91.00	0.458
OFC_R	0.0001	0.0079	−0.0017	0.0087	−0.0016	0.548	−0.0018	0.0054	0.0019	0.0128	114.00	0.045^*^
FPC_L	0.0028	0.0055	−0.0017	0.0043	−0.0061	0.006^**^	−0.0025	0.0053	0.0046	0.0060	137.00	0.001^**^
FPC_R	0.0011	0.0060	−0.0009	0.0055	−0.0029	0.145	0.0003	0.0039	0.0036	0.0032	115.00	0.039^*^
**DSB**												
DLPFC_L	0.0003	0.0023	0.0003	0.0018	−0.0004	0.611	−0.0004	0.0019	−0.0008	0.0050	65.00	0.560
DLPFC_R	0.0001	0.0038	−0.0003	0.0045	0.0001	0.927	0.0013	0.0074	−0.0020	0.0092	61.00	0.426
VLPFC_L	0.0006	0.0073	−0.0027	0.0074	−0.0027	0.298	−0.0025	0.0030	−0.0012	0.0049	89.00	0.136
VLPFC_R	0.0008	0.0047	0.0033	0.0094	0.0005	0.927	0.0040	0.0095	0.0021	0.0069	55.00	0.475
OFC_L	−0.0002	0.0023	−0.0001	0.0076	0.0004	0.706	0.0012	0.0026	−0.0035	0.0035	42.00	0.095
OFC_R	0.0016	0.0077	0.0009	0.0066	0.0007	0.747	0.0003	0.0045	−0.0009	0.0047	60.00	0.396
FPC_L	0.0001	0.0013	0.0016	0.0046	0.0014	0.517	0.0000	0.0035	−0.0018	0.0034	58.00	0.339
FPC_R	0.0004	0.0028	0.0006	0.0044	0.0006	0.517	0.0017	0.0037	0.0010	0.0048	44.00	0.085

^*^p < 0.05 and ^**^p < 0.01.

As shown in Figure 5, the HbO_2_ signal beta amplitude in PFC tended to increase in VFT and decrease in Stroop task at the 6-month follow-up compared to that at baseline among the participants with MCI. No significant difference was observed when comparing the HbO_2_ beta coefficient between baseline and the 6-month follow-up. When comparing the changes from baseline in both groups, the observed prefrontal activity during the Stroop test at the 6-month follow-up was similar in both groups (Table 5).

As illustrated in Figure 6, the activation level measured by HbR beta coefficient tended to decrease over time during performance of the Stroop task. In the left DLPFC and FPC, the HbR beta significantly decreased (hence, overall HbR level being significantly increased) at the 6-month follow-up compared to those at baseline in participants with MCI. When comparing the changes from baseline in both groups, the changes in participants with MCI were greater than those in healthy controls in left and right FPC regions (Table 6).

## 4 Discussion

### 4.1 Summary of the main findings

This study aimed to investigate improvements in cognitive function and underlying mechanisms after application of a community-based dementia prevention program involving herbal medicine and acupuncture in older individuals with MCI who reside in Busan, Republic of Korea. To explore potential therapeutic mechanisms, fNIRS was utilized to measure changes in HbO_2_ and HbR beta coefficient in prefrontal regions during performance of cognitive tasks. The major findings indicate that the community-based dementia prevention program used in this study was effective in improving cognitive assessment scores, such as the MoCA and CIST, and performance in cognitive tasks, over the 6-month study period. Furthermore, the HbR beta values in the left DLPFC and left FPC during the Stroop task were lower than that at baseline at the 6-month follow-up in patients with MCI, and this difference was also significant compared to healthy controls.

### 4.2 Improvement in cognitive function after application of the community-based program

Community-based participatory research is a collaborative approach in which the community and academic partners collaborate to formulate research questions, implement research activities, and disseminate findings. This approach allows researchers to gain a deeper understanding of the strengths, challenges, and opportunities within the community, thereby shedding light on the social, physical, and policy factors that affect community health, which may enhance translation of the research findings into practice (41). Academic researchers thus remain committed to the community throughout the research process and beyond (42). In our earlier study, which was conducted as part of the Dementia Prevention & Care Program administered by the Busan Association of Korean Medicine, as community stakeholder, we observed cognitive benefits from herbal medication and acupuncture interventions in 229 elderly individuals diagnosed with MCI. After 6 months of program participation, we noted improvements in cognitive function among participants with MCI, and the satisfaction scores of the participants were predominantly high (6). These findings are consistent with those of the current research implemented by our academic researcher (6, 7).

Our decision to focus on MCI was influenced by the distinctive demographic profile of the Busan community, which is recognized as having the most rapidly aging population in the Republic of Korea (43). In this study, we used fNIRS to explore the therapeutic mechanisms underlying these favorable outcomes. Through our community-centered approach, we gained insights into the use of herbal medicine and acupuncture by older adults with MCI residing in Busan. By implementing effective interventions via the 2021 Dementia Prevention & Care Program, we strived to address cognitive impairment in the older population of the Busan community. These findings may serve as a foundation for future clinical applications.

### 4.3 Exploring potential therapeutic mechanisms via hemodynamic responses

Recently, neuroimaging studies investigating hemodynamic changes associated with cognitive impairment have reported blunted responses in the frontal cortex in dementia, whereas findings in MCI have been more varied, with some studies suggesting hyperactivated hemodynamic responses as a compensatory mechanism (44). Another study showed that in patients with MCI, the herbal medicine group showed improved memory measurements over time compared to the placebo group. Brain activation in drug groups with fMRI was decreased in DLPFC and increased in medial prefrontal, parietal, and temporal cortex (8). These conflicting results in MCI may stem from differences in the cognitive tasks and regions of interest (ROI). To date, clinical studies evaluating the efficacy and hemodynamic responses to traditional medicine including herbal medicines and acupuncture for MCI are limited. In this study, we aimed to investigate the therapeutic mechanisms underlying the effectiveness of herbal medicine plus acupuncture therapy in MCI by measuring changes in hemodynamic responses in the PFC using fNIRS during cognitive tasks. We expected that PFC activation during cognitive tasks in patients with MCI would vary according to ROI or task type over time after intervention. We observed stronger HbO_2_ beta coefficient in the PFC during the VFT in patients with MCI at the 3-month follow-up with intervention. While a trend toward increased HbO_2_ beta coefficient during the Stroop task was observed in the MCI group at the 3-month follow-up, this beta value decreased at the 6-month follow-up, resembling the average response of healthy controls at 6 months (Figure 5). Moreover, HbR beta coefficient during the Stroop task tended to decrease over time, especially the HbR beta in the left DLPFC and FPC significantly decreased at the 6-month follow-up compared to those at baseline in participants with MCI (Figure 6, Table 6). Therefore, PFC activation decreased during Stroop task after intervention in patients with MCI. This phenomenon, in which the hemodynamic response decreases with improvement in cognitive performance during follow-up visits, is consistent with a previous study on physically active individuals with MCI over a 1-year period, and they judged this tendency to be more efficient in cortical oxygenation (45).

With regard to DLPFC and FPC that showed statistically significant brain activity reduction in this study, the association can be found in previous studies. Consistent with our study, previous studies have demonstrated decreased activation of DLPFC in herbal medicine groups and increased in placebo groups in patients with MCI (8). Meanwhile, the FPC is known to correlate with insight loss, defined as a lack of awareness of mental symptoms due to frank denial or unconcern for their consequences, in frontotemporal dementia and Alzheimer's disease (46). Additionally, the Stroop task, known for the conflict adaptation effect, involves the FPC, as reported by Lee and Kim (47). The authors suggested that the FPC plays a critical role in conflict regulation through recruitment of higher cognitive control strategies, along with the anterior cingulate cortex and DLPFC. Another investigation into the functional mechanisms of Stroop interference and reverse-Stroop interference effects using functional magnetic resonance imaging (fMRI) suggested that the PFC and cingulate cortex exhibit greater sensitivity to reverse-Stroop tasks than to Stroop tasks (30). In our study, the Stroop task comprised hard and easy components corresponding to the reverse-Stroop and Stroop tasks, respectively. Notably, during the reverse-Stroop task, compared to the Stroop task, HbR beta coefficient in the left FPC and left DLPFC significantly decreased in MCI after 6 months of combination therapy. Additionally, HbR beta coefficient in the right OFC and FPC were significantly lower in the MCI group than in healthy controls. In previous studies, the FPC region has been identified to play a key role in higher-order and intricate cognitive processes (47–49). Based on these preliminary findings, we propose that herbal medicine and acupuncture may enhance cognitive function in MCI by improving efficient the FPC region.

### 4.4 Herbal medicines used in this community-based dementia prevention program

In the Dementia Prevention & Care Program in Busan, herbal medicine was used based on previous reports of cognitive improvement. Several studies have indicated that oral administration of certain herbal medicines for MCI may help prevent progression into dementia and improve cognitive function (5, 50). An observational study in Korea investigated the effectiveness of herbal medicines, including Yukmijihwang-tang, Samhwangsasim-tang, Palmul-tang, Banhasasim-tang, and Yukgunja-tang, against cognitive impairment, based on pattern identification diagnosis. These herbal medicines have the potential to improve cognitive function in patients with MCI, with Samhwangsasim-tang and Palmul-tang being frequently prescribed (51). In this study, the effectiveness of combined acupuncture and herbal medicines, including Kami Guibi-tang, Yukmijihwang-tang, and Dangguijagyag-san, was investigated in older individuals with MCI living in Busan in the Republic of Korea. KamiGuibi-tang, Yukmijihwang-tang, and Dangguijagyag-san were the most frequently used herbal medicines. A recent randomized controlled trial on the efficacy of Kami Guibi-tang for amnestic MCI showed that the scores on the Clinical Dementia Rating Scale-Sum of Boxes (CDR-SB) instrument improved significantly in the Kami Guibi-tang group compared with those in the placebo group (17). Additionally, Yukmijihwang-tang, which has been shown to improve cognitive impairment in observational studies (51), ameliorated hippocampal memory impairment in a chronic restraint stress mouse model (19).

### 4.5 Study limitations and future directions

Our study has some limitations. The most significant limitation is that since this was a retrospective observational study, an MCI control group without intervention was not included. This study was not conducted in a prospective, strict clinical trial setting; rather, it evaluated the overall effects of a community-based program in a real-world setting. While the clinicians voluntarily formulated the research questions and conducted the study during the implementation of the program, future research should include prospective randomized placebo-controlled trial-based studies with patient controls to rigorously explore the potential underlying mechanisms. Second, as various interventions, including herbal medicine, acupuncture, and pharmacopuncture, were used in the community-based Dementia Prevention & Care Program, it was not possible to determine the specific efficacy of each intervention. The combined effect of the program observed in this study reflects the real-world settings in which combined interventions are performed in clinics. Third, this was a preliminary study measured PFC hemodynamics using fNIRS in a small number of participants, with the healthy control group being about half the size of MCI group. The fNIRS measurement schedule differed between groups, primarily comparing hemodynamic responses after 6 months in both groups, with an additional 3-month measurement for the MCI group to observe longitudinal changes. Lastly, hemodynamic analysis using fNIRS was limited to the PFC. fNIRS allows measurements from a limited number of channels and only in the cortex. In the future, it will be necessary to observe the entire brain, including the subcortical areas, using neuroimaging techniques such as fMRI.

## 5 Conclusions

The present study provides preliminary evidence supporting the effectiveness of herbal medicine and acupuncture in improving cognitive function in patients diagnosed with MCI. Specifically, we observed significant decrease in the HbR beta coefficient in the left DLPFC and FPC, particularly during the Stroop task. This change in prefrontal activation may indicate more efficient cortical oxygenation, potentially reflecting an underlying mechanism of cognitive improvement. These findings hold promise for the development of community-based dementia prevention and management strategies for older adults experiencing cognitive impairment.

## Data Availability

The raw data supporting the conclusions of this article will be made available by the authors, without undue reservation.
