# Supplementary material for: Effectiveness of Korean medicine treatments in improving cognitive function and prefrontal cortex activity in older individuals with mild cognitive impairment: retrospective observational study
Source: Front Neurol. 2024 Oct 30;15:1440111. doi: 10.3389/fneur.2024.1440111 (PMC11557411; doi:10.3389/fneur.2024.1440111)
Supplement: Supplementary file 1 [file Table_1.DOCX]

Supplementary Material

**
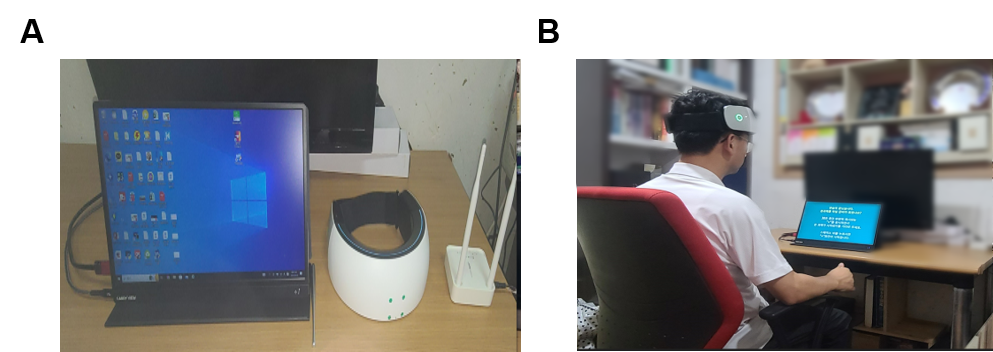
**

**Supplementary Figure 1.** (A) fNIRS instrument setup and (B) fNIRS measurement during cognitive tasks
